# 2-(2,6-Di­chloro­phen­yl)-1-pentyl-4,5-diphenyl-1*H*-imidazole

**DOI:** 10.1107/S1600536813011446

**Published:** 2013-05-11

**Authors:** Mehmet Akkurt, Shaaban K. Mohamed, Kuldip Singh, Adel A. Marzouk, Antar A. Abdelhamid

**Affiliations:** a Department of Physics, Faculty of Sciences, Erciyes University, 38039 Kayseri, Turkey; b Chemistry and Environmental Division, Manchester Metropolitan University, Manchester M1 5GD, England; c Chemistry Department, Faculty of Sccience, Mini University, 61519 El-Minia, Egypt; d Department of Chemistry, University of Leicester, Leicester, England; e Pharmaceutical Chemistry Department, Faculty of Pharmacy, Al Azhar University, Egypt

## Abstract

The title compound, C_26_H_24_Cl_2_N_2_, crystallizes with two independent mol­ecules (1 and 2) in the asymmetric unit. In mol­ecule 1, the two phenyl and 2,6-di­chloro­phenyl rings are inclined to the imidazole ring at angles of 74.12 (14), 26.13 (14) and 67.30 (14)°, respectively. In mol­ecule 2, due to the different mol­ecular environment in the crystal, the corresponding angles are different, *viz.* 71.72 (15), 16.14 (15) and 80.41 (15)°, respectively. In the crystal, mol­ecules 1 and 2 are linked by C—H⋯Cl inter­actions, and inversion-related 2 mol­ecules are linked by C—H⋯π inter­actions. There are no other significant inter­molecular inter­actions present.

## Related literature

For some biological applications of imidazoles, see: Prabhu & Radha (2012 ▶); Sharma *et al.* (2009 ▶, 2010 ▶); Pandey *et al.* (2009 ▶); Sisko & Mellinger (2002 ▶); Puratchikody & Doble (2007 ▶). For the synthesis of imidazole-containing compounds and a similar structure, see: Simpson *et al.* (2013 ▶).
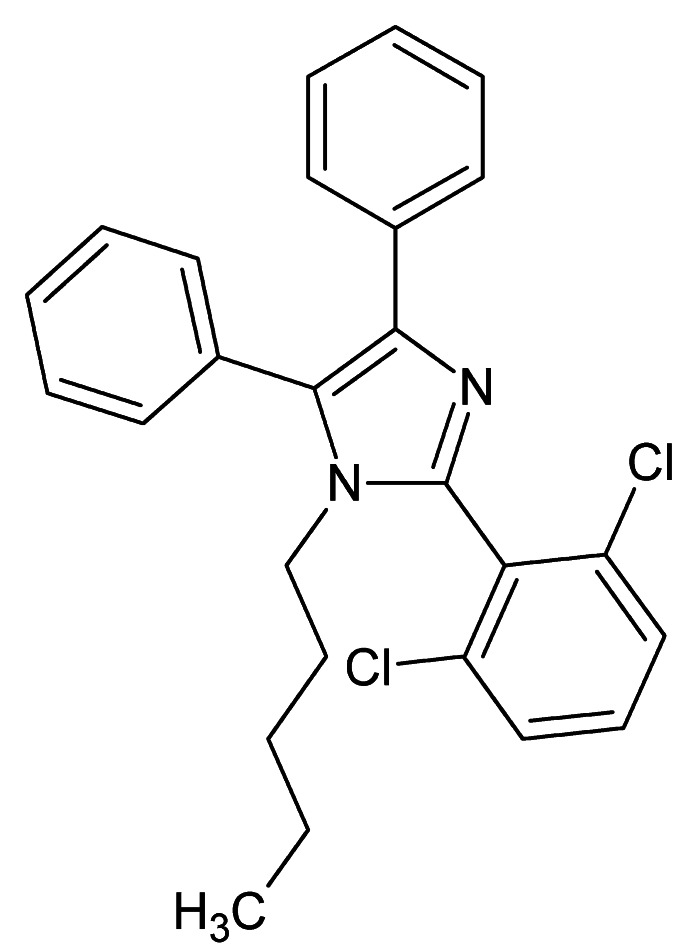


## Experimental

### 

#### Crystal data

C_26_H_24_Cl_2_N_2_
*M* *_r_* = 435.37
Monoclinic, 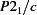

*a* = 20.172 (6) Å
*b* = 15.947 (5) Å
*c* = 14.500 (5) Å
β = 105.293 (7)°
*V* = 4499 (2) Å^3^
*Z* = 8
Mo *K*α radiation
μ = 0.30 mm^−1^
*T* = 150 K
0.34 × 0.16 × 0.11 mm

#### Data collection

Bruker APEX 2000 CCD area-detector diffractometer
Absorption correction: multi-scan (*SADABS*; Bruker, 2011 ▶) *T*_min_ = 0.516, *T*_max_ = 0.928
34900 measured reflections
8837 independent reflections
5052 reflections with *I* > 2σ(*I*)
*R*_int_ = 0.123

#### Refinement

*R*[*F* ^2^ > 2σ(*F* ^2^)] = 0.054
*wR*(*F* ^2^) = 0.098
*S* = 0.84
8837 reflections
543 parameters
H-atom parameters constrained
Δρ_max_ = 0.29 e Å^−3^
Δρ_min_ = −0.39 e Å^−3^

### 

Data collection: *SMART* (Bruker, 2011 ▶); cell refinement: *SAINT* (Bruker, 2011 ▶); data reduction: *SAINT*; program(s) used to solve structure: *SHELXS97* (Sheldrick, 2008 ▶); program(s) used to refine structure: *SHELXL97* (Sheldrick, 2008 ▶); molecular graphics: *ORTEP-3 for Windows* (Farrugia, 2012 ▶); software used to prepare material for publication: *WinGX* (Farrugia, 2012 ▶) and *PLATON* (Spek, 2009 ▶).

## Supplementary Material

Crystal structure: contains datablock(s) global, I. DOI: 10.1107/S1600536813011446/su2592sup1.cif

Structure factors: contains datablock(s) I. DOI: 10.1107/S1600536813011446/su2592Isup2.hkl

Supplementary material file. DOI: 10.1107/S1600536813011446/su2592Isup3.cml

Additional supplementary materials:  crystallographic information; 3D view; checkCIF report

## Figures and Tables

**Table 1:** Hydrogen-bond geometry (Å, °) *Cg*1 is the centroid of the N1/N2/C1–C3 imidazole ring.

*D*—H⋯*A*	*D*—H	H⋯*A*	*D*⋯*A*	*D*—H⋯*A*
C12*A*—H12*A*⋯Cl1^i^	0.95	2.70	3.512 (4)	143
C21—H21⋯*Cg*1^ii^	0.95	2.88	3.734 (3)	151

Symmetry codes: (i) 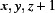; (ii) 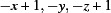.

**Fig. 1.**
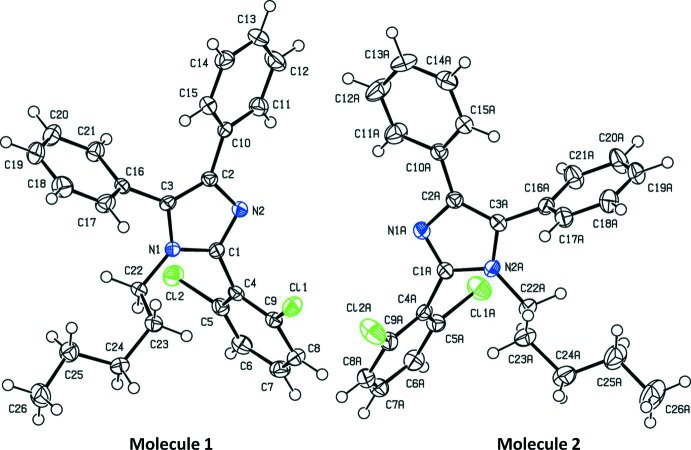
The molecular structure of the two independent molecules (1 and 2-with suffix A) of the title compound, with atom labelling. Displacement ellipsoids are drawn at the 50% probability level.

**Fig. 2.**
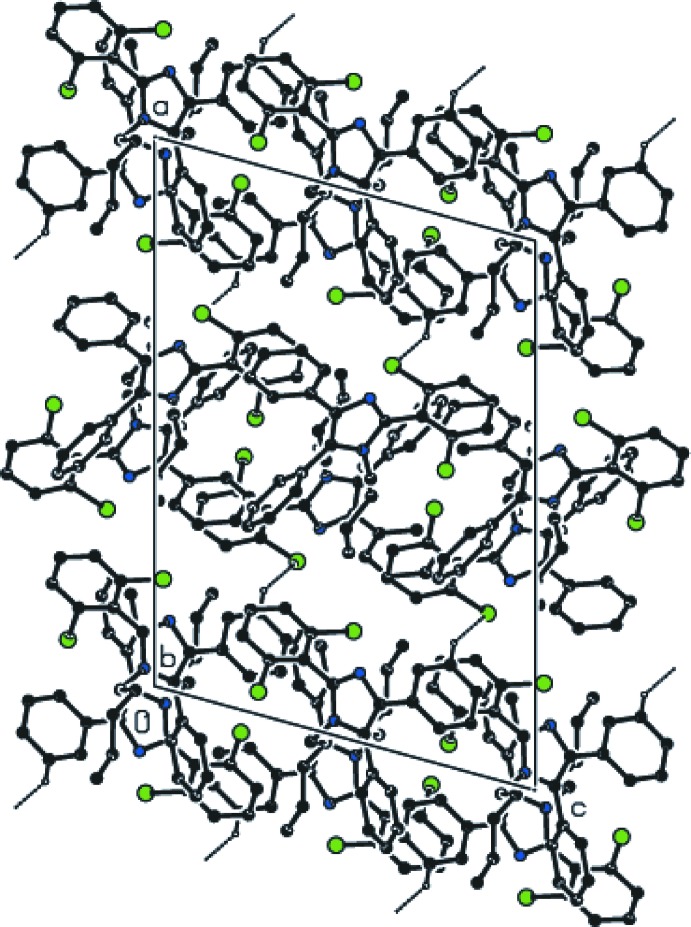
Crystal packing of the title compound viewed along the b axis. Hydrogen bonds are drawn as dashed lines (see Table 1 for details).

## supplementary crystallographic information

### Comment

Imidazoles are considered as an important pharmacophore in medicinal chemistry encompassing wide spectrum of biological activities (Prabhu & Radha, 2012) such as antibacterial, antiviral (Sharma *et al.*, 2009, 2010), antirheumatoid arthritis, anticancer (Sisko & Mellinger, 2002), antitubercular (Pandey *et al.*, 2009) and anti-inflammatory (Puratchikody & Doble, 2007). The title compound has been synthesized among series of imidazole derivatives according to our on going study in green synthesis of multi-substituted imidazoles *via* a multicomponent reactions method using ionic liquid as a recyclable catalyst.

In the title compound (Fig. 1), the asymmetric unit contains two independent molecules; 1 (with N1) and 2 (with N1A). In molecule 1, the two phenyl (C10–C15 and C16–C21) and 2,6-dichlorophenyl (C4–C9) rings are inclined to the N1/N2/C1–C3 imidazole ring at angles of 74.12 (14), 26.13 (14) and 67.30 (14)°, respectively. In molecule 2, the corresponding angles are 71.72 (15), 16.14 (15) and 80.41 (15)°, respectively (the atom labels of molecule 2 are with the extra suffix A). The differences between the corresponding angles arises due to the intra- and intermolecular interactions of the different molecular environments. They are different from those reported in a similar structure (Simpson *et al.*, 2013).

In the crystal, molecules 1 and 2 are linked by C—H···Cl interactions, and inversion related 2 molecules are linked by C—H···π interactions (Table 1 and Fig. 2). There are no other significant intermolecular interactions present.

### Experimental

The title compound was synthesized following the previously reported procedure (Simpson *et al.*, 2013). Block-like colourless crystals, suitable for X-ray diffraction analysis, were obtained by slow evaporation of an ethanol solution of the title compound.

### Refinement

All H-atoms were included in calculated positions and refined using a riding model: C—H = 0.95, 0.99 and 0.98 Å, for CH(aromatic), CH_2_ and CH_3_ H atoms, respectively, with *U*_iso_(H) = 1.5*U*_eq_(C-methyl) and = 1.2*U*_eq_(C) for other H atoms. One reflection (1 0 0) has been omitted in the final refinement cycles.

### Crystal data

**Table:**

C_26_H_24_Cl_2_N_2_	*F*(000) = 1824
*M**_r_* = 435.37	*D*_x_ = 1.285 Mg m^−^^3^
Monoclinic, *P*2_1_/*c*	Mo *K*α radiation, λ = 0.71073 Å
Hall symbol: -P 2ybc	Cell parameters from 792 reflections
*a* = 20.172 (6) Å	θ = 2.5–24.2°
*b* = 15.947 (5) Å	µ = 0.30 mm^−^^1^
*c* = 14.500 (5) Å	*T* = 150 K
β = 105.293 (7)°	Block, colourless
*V* = 4499 (2) Å^3^	0.34 × 0.16 × 0.11 mm
*Z* = 8	

### Data collection

**Table:**

Bruker APEX 2000 CCD area-detector diffractometer	8837 independent reflections
Radiation source: fine-focus sealed tube	5052 reflections with *I* > 2σ(*I*)
Graphite monochromator	*R*_int_ = 0.123
phi and ω scans	θ_max_ = 26.0°, θ_min_ = 1.7°
Absorption correction: multi-scan (*SADABS*; Bruker, 2011)	*h* = −24→24
*T*_min_ = 0.516, *T*_max_ = 0.928	*k* = −19→19
34900 measured reflections	*l* = −17→17

### Refinement

**Table:**

Refinement on *F*^2^	Primary atom site location: structure-invariant direct methods
Least-squares matrix: full	Secondary atom site location: difference Fourier map
*R*[*F*^2^ > 2σ(*F*^2^)] = 0.054	Hydrogen site location: inferred from neighbouring sites
*wR*(*F*^2^) = 0.098	H-atom parameters constrained
*S* = 0.84	*w* = 1/[σ^2^(*F*_o_^2^) + (0.0212*P*)^2^] where *P* = (*F*_o_^2^ + 2*F*_c_^2^)/3
8837 reflections	(Δ/σ)_max_ = 0.001
543 parameters	Δρ_max_ = 0.29 e Å^−^^3^
0 restraints	Δρ_min_ = −0.39 e Å^−^^3^

### Special details

**Table:**

Geometry. Bond distances, angles *etc*. have been calculated using the rounded fractional coordinates. All su's are estimated from the variances of the (full) variance-covariance matrix. The cell e.s.d.'s are taken into account in the estimation of distances, angles and torsion angles
Refinement. Refinement on *F*^2^ for ALL reflections except those flagged by the user for potential systematic errors. Weighted *R*-factors *wR* and all goodnesses of fit *S* are based on *F*^2^, conventional *R*-factors *R* are based on *F*, with *F* set to zero for negative *F*^2^. The observed criterion of *F*^2^ > σ(*F*^2^) is used only for calculating -*R*-factor-obs *etc*. and is not relevant to the choice of reflections for refinement. *R*-factors based on *F*^2^ are statistically about twice as large as those based on *F*, and *R*-factors based on ALL data will be even larger.

### Fractional atomic coordinates and isotropic or equivalent isotropic displacement parameters (Å^2^)

**Table:**

	*x*	*y*	*z*	*U*_iso_*/*U*_eq_	
Cl1	0.29823 (3)	0.30921 (5)	0.37517 (5)	0.0297 (3)	
Cl2	0.46202 (4)	0.10547 (5)	0.23512 (5)	0.0328 (2)	
N1	0.46666 (10)	0.19189 (13)	0.45220 (15)	0.0205 (7)	
N2	0.36928 (10)	0.11983 (13)	0.43427 (14)	0.0204 (8)	
C1	0.40305 (13)	0.17285 (16)	0.39371 (18)	0.0200 (9)	
C2	0.41270 (13)	0.10305 (16)	0.52403 (18)	0.0195 (9)	
C3	0.47296 (13)	0.14686 (16)	0.53614 (18)	0.0202 (9)	
C4	0.37540 (13)	0.20741 (17)	0.29671 (18)	0.0210 (9)	
C5	0.39632 (13)	0.17906 (17)	0.21808 (19)	0.0241 (9)	
C6	0.36586 (14)	0.20696 (18)	0.1261 (2)	0.0289 (10)	
C7	0.31307 (14)	0.26443 (18)	0.1118 (2)	0.0295 (10)	
C8	0.29128 (14)	0.29496 (17)	0.18758 (19)	0.0274 (10)	
C9	0.32282 (13)	0.26701 (17)	0.27873 (18)	0.0227 (9)	
C10	0.38985 (13)	0.04658 (17)	0.58975 (18)	0.0213 (9)	
C11	0.34051 (14)	−0.01395 (18)	0.5538 (2)	0.0298 (10)	
C12	0.31617 (15)	−0.06537 (19)	0.6145 (2)	0.0382 (11)	
C13	0.34109 (15)	−0.05737 (19)	0.7124 (2)	0.0348 (11)	
C14	0.39050 (14)	0.00240 (18)	0.7488 (2)	0.0308 (11)	
C15	0.41468 (14)	0.05382 (17)	0.68861 (19)	0.0264 (10)	
C16	0.53813 (13)	0.14413 (17)	0.61316 (18)	0.0203 (9)	
C17	0.56398 (14)	0.21282 (18)	0.67047 (19)	0.0296 (10)	
C18	0.62524 (15)	0.20745 (19)	0.7407 (2)	0.0346 (11)	
C19	0.66190 (15)	0.1337 (2)	0.7538 (2)	0.0365 (11)	
C20	0.63697 (14)	0.0648 (2)	0.6983 (2)	0.0356 (11)	
C21	0.57527 (14)	0.07020 (18)	0.62853 (19)	0.0280 (10)	
C22	0.51523 (13)	0.25222 (16)	0.43213 (19)	0.0226 (9)	
C23	0.49632 (14)	0.34200 (17)	0.4496 (2)	0.0289 (10)	
C24	0.54602 (13)	0.40739 (17)	0.43014 (19)	0.0287 (10)	
C25	0.61807 (13)	0.40211 (18)	0.49662 (19)	0.0317 (10)	
C26	0.66054 (15)	0.47987 (19)	0.4898 (2)	0.0425 (11)	
Cl1A	0.04153 (4)	0.60347 (5)	0.77291 (5)	0.0408 (3)	
Cl2A	0.19909 (4)	0.82158 (5)	1.02289 (5)	0.0462 (3)	
N1A	0.12565 (11)	0.63396 (14)	1.03916 (15)	0.0237 (8)	
N2A	0.02673 (11)	0.69733 (14)	0.97245 (15)	0.0240 (8)	
C1A	0.09269 (14)	0.68241 (17)	0.96875 (19)	0.0229 (9)	
C2A	0.07903 (13)	0.61583 (17)	1.09207 (18)	0.0230 (9)	
C3A	0.01745 (13)	0.65406 (17)	1.05079 (18)	0.0229 (9)	
C4A	0.12283 (13)	0.71366 (17)	0.89307 (18)	0.0227 (9)	
C5A	0.10486 (14)	0.68040 (17)	0.80107 (19)	0.0272 (10)	
C6A	0.13600 (14)	0.70545 (18)	0.7313 (2)	0.0312 (10)	
C7A	0.18707 (14)	0.76503 (18)	0.7527 (2)	0.0316 (11)	
C8A	0.20691 (14)	0.79964 (18)	0.84299 (19)	0.0307 (10)	
C9A	0.17463 (14)	0.77444 (18)	0.91125 (19)	0.0261 (9)	
C10A	0.10028 (14)	0.56404 (17)	1.17776 (19)	0.0235 (9)	
C11A	0.16101 (15)	0.51845 (19)	1.1938 (2)	0.0374 (11)	
C12A	0.18591 (17)	0.4736 (2)	1.2769 (2)	0.0458 (12)	
C13A	0.14991 (16)	0.47077 (19)	1.3459 (2)	0.0400 (11)	
C14A	0.08958 (15)	0.51536 (18)	1.3310 (2)	0.0333 (11)	
C15A	0.06511 (14)	0.56180 (17)	1.24883 (19)	0.0280 (10)	
C16A	−0.05062 (13)	0.65026 (18)	1.07212 (18)	0.0241 (9)	
C17A	−0.07410 (14)	0.71327 (19)	1.1209 (2)	0.0324 (11)	
C18A	−0.13809 (15)	0.7079 (2)	1.1390 (2)	0.0375 (11)	
C19A	−0.17886 (15)	0.6386 (2)	1.1090 (2)	0.0376 (11)	
C20A	−0.15554 (15)	0.5749 (2)	1.0622 (2)	0.0429 (11)	
C21A	−0.09166 (15)	0.58025 (19)	1.0439 (2)	0.0364 (11)	
C22A	−0.02348 (14)	0.75371 (17)	0.91036 (19)	0.0285 (10)	
C23A	−0.01743 (15)	0.84245 (18)	0.9496 (2)	0.0372 (11)	
C24A	−0.07254 (15)	0.9019 (2)	0.8910 (2)	0.0421 (12)	
C25A	−0.14530 (15)	0.8789 (2)	0.8894 (2)	0.0483 (14)	
C26A	−0.19694 (17)	0.9481 (2)	0.8481 (2)	0.0665 (17)	
H6	0.38120	0.18670	0.07360	0.0350*	
H7	0.29150	0.28320	0.04890	0.0350*	
H8	0.25500	0.33480	0.17730	0.0330*	
H11	0.32320	−0.02030	0.48660	0.0360*	
H12	0.28220	−0.10640	0.58870	0.0460*	
H13	0.32440	−0.09260	0.75420	0.0420*	
H14	0.40800	0.00820	0.81610	0.0370*	
H15	0.44870	0.09470	0.71480	0.0320*	
H17	0.53920	0.26410	0.66120	0.0360*	
H18	0.64200	0.25460	0.77990	0.0420*	
H19	0.70450	0.13030	0.80130	0.0440*	
H20	0.66200	0.01370	0.70790	0.0430*	
H21	0.55820	0.02240	0.59060	0.0340*	
H22A	0.51650	0.24620	0.36470	0.0270*	
H22B	0.56180	0.23980	0.47320	0.0270*	
H23A	0.49450	0.34730	0.51680	0.0350*	
H23B	0.44980	0.35400	0.40830	0.0350*	
H24A	0.54890	0.40090	0.36340	0.0340*	
H24B	0.52720	0.46390	0.43600	0.0340*	
H25A	0.64150	0.35190	0.48010	0.0380*	
H25B	0.61500	0.39560	0.56330	0.0380*	
H26A	0.66320	0.48700	0.42380	0.0640*	
H26B	0.70700	0.47330	0.53200	0.0640*	
H26C	0.63880	0.52930	0.50940	0.0640*	
H6A	0.12220	0.68170	0.66910	0.0370*	
H7A	0.20870	0.78240	0.70510	0.0380*	
H8A	0.24250	0.84040	0.85810	0.0370*	
H11A	0.18580	0.51830	1.14650	0.0450*	
H12A	0.22820	0.44420	1.28680	0.0550*	
H13A	0.16650	0.43870	1.40250	0.0480*	
H14A	0.06450	0.51410	1.37800	0.0400*	
H15A	0.02370	0.59270	1.24040	0.0340*	
H17A	−0.04600	0.76090	1.14230	0.0390*	
H18A	−0.15380	0.75190	1.17210	0.0450*	
H19A	−0.22300	0.63490	1.12060	0.0450*	
H20A	−0.18340	0.52680	1.04230	0.0520*	
H21A	−0.07590	0.53570	1.01170	0.0440*	
H22C	−0.07050	0.73240	0.90470	0.0340*	
H22D	−0.01590	0.75420	0.84560	0.0340*	
H23C	0.02850	0.86480	0.95060	0.0450*	
H23D	−0.02110	0.84100	1.01630	0.0450*	
H24C	−0.06310	0.95920	0.91740	0.0500*	
H24D	−0.06860	0.90320	0.82440	0.0500*	
H25C	−0.14750	0.86630	0.95540	0.0580*	
H25D	−0.15840	0.82740	0.85080	0.0580*	
H26D	−0.18670	0.99780	0.88920	0.1000*	
H26E	−0.24350	0.92850	0.84490	0.1000*	
H26F	−0.19380	0.96250	0.78370	0.1000*	

### Atomic displacement parameters (Å^2^)

**Table:**

	*U*^11^	*U*^22^	*U*^33^	*U*^12^	*U*^13^	*U*^23^
Cl1	0.0307 (4)	0.0309 (5)	0.0279 (4)	0.0085 (4)	0.0082 (3)	0.0019 (3)
Cl2	0.0339 (4)	0.0290 (4)	0.0374 (4)	0.0071 (4)	0.0127 (3)	0.0001 (4)
N1	0.0176 (12)	0.0162 (13)	0.0271 (13)	0.0001 (10)	0.0047 (10)	0.0034 (11)
N2	0.0194 (12)	0.0186 (14)	0.0236 (13)	−0.0003 (11)	0.0063 (10)	0.0013 (10)
C1	0.0191 (15)	0.0162 (16)	0.0250 (15)	0.0016 (13)	0.0063 (12)	0.0001 (13)
C2	0.0213 (15)	0.0159 (15)	0.0224 (15)	0.0006 (13)	0.0075 (12)	0.0009 (13)
C3	0.0216 (16)	0.0148 (16)	0.0245 (15)	0.0042 (13)	0.0064 (12)	0.0018 (12)
C4	0.0199 (15)	0.0190 (16)	0.0240 (15)	−0.0055 (13)	0.0059 (12)	0.0018 (13)
C5	0.0238 (16)	0.0192 (17)	0.0301 (16)	−0.0030 (13)	0.0087 (13)	−0.0024 (13)
C6	0.0330 (18)	0.0276 (19)	0.0268 (16)	−0.0058 (15)	0.0093 (14)	−0.0037 (14)
C7	0.0335 (18)	0.0290 (19)	0.0236 (16)	−0.0049 (15)	0.0031 (14)	0.0052 (14)
C8	0.0261 (17)	0.0247 (18)	0.0296 (17)	0.0029 (14)	0.0042 (13)	0.0052 (14)
C9	0.0224 (16)	0.0199 (17)	0.0265 (16)	−0.0022 (13)	0.0076 (13)	0.0015 (13)
C10	0.0173 (15)	0.0182 (16)	0.0290 (16)	0.0042 (13)	0.0073 (12)	0.0042 (13)
C11	0.0288 (18)	0.0318 (19)	0.0273 (17)	−0.0042 (15)	0.0048 (14)	0.0021 (14)
C12	0.0352 (19)	0.036 (2)	0.044 (2)	−0.0140 (16)	0.0117 (16)	0.0020 (16)
C13	0.0347 (19)	0.036 (2)	0.0376 (19)	−0.0047 (16)	0.0167 (15)	0.0106 (16)
C14	0.0308 (18)	0.035 (2)	0.0269 (17)	0.0040 (15)	0.0083 (14)	0.0052 (15)
C15	0.0231 (16)	0.0275 (18)	0.0296 (17)	−0.0016 (14)	0.0085 (13)	0.0018 (14)
C16	0.0182 (15)	0.0195 (16)	0.0250 (15)	−0.0015 (13)	0.0088 (12)	0.0016 (13)
C17	0.0318 (18)	0.0206 (18)	0.0343 (18)	0.0033 (14)	0.0050 (14)	0.0002 (14)
C18	0.0360 (19)	0.030 (2)	0.0335 (18)	−0.0097 (16)	0.0015 (15)	−0.0054 (15)
C19	0.0249 (17)	0.046 (2)	0.0322 (18)	−0.0039 (16)	−0.0038 (14)	0.0039 (16)
C20	0.0259 (18)	0.035 (2)	0.0420 (19)	0.0063 (15)	0.0019 (15)	0.0047 (16)
C21	0.0266 (17)	0.0257 (18)	0.0305 (17)	0.0022 (14)	0.0052 (14)	−0.0029 (14)
C22	0.0209 (16)	0.0213 (17)	0.0270 (16)	−0.0014 (13)	0.0089 (13)	0.0058 (13)
C23	0.0248 (17)	0.0226 (18)	0.0400 (18)	0.0002 (14)	0.0096 (14)	0.0059 (14)
C24	0.0289 (17)	0.0221 (17)	0.0339 (17)	0.0019 (14)	0.0064 (14)	0.0057 (14)
C25	0.0293 (17)	0.0292 (19)	0.0337 (17)	−0.0020 (15)	0.0031 (14)	−0.0012 (15)
C26	0.0318 (19)	0.034 (2)	0.058 (2)	−0.0081 (16)	0.0054 (16)	−0.0036 (17)
Cl1A	0.0438 (5)	0.0379 (5)	0.0407 (5)	−0.0129 (4)	0.0112 (4)	−0.0081 (4)
Cl2A	0.0511 (5)	0.0586 (6)	0.0299 (4)	−0.0256 (5)	0.0126 (4)	−0.0098 (4)
N1A	0.0219 (13)	0.0264 (15)	0.0236 (13)	−0.0004 (11)	0.0072 (10)	0.0015 (11)
N2A	0.0204 (13)	0.0262 (15)	0.0248 (13)	0.0044 (11)	0.0048 (10)	0.0041 (11)
C1A	0.0213 (16)	0.0221 (17)	0.0258 (16)	−0.0012 (13)	0.0072 (13)	0.0010 (13)
C2A	0.0234 (16)	0.0216 (17)	0.0248 (15)	0.0002 (14)	0.0079 (13)	0.0002 (13)
C3A	0.0221 (16)	0.0225 (17)	0.0247 (15)	−0.0027 (13)	0.0070 (13)	−0.0002 (13)
C4A	0.0207 (16)	0.0244 (17)	0.0236 (15)	0.0066 (13)	0.0070 (12)	0.0047 (13)
C5A	0.0283 (17)	0.0236 (18)	0.0302 (17)	−0.0003 (14)	0.0085 (14)	0.0016 (14)
C6A	0.0335 (18)	0.036 (2)	0.0250 (16)	0.0035 (16)	0.0093 (14)	−0.0014 (14)
C7A	0.0291 (18)	0.034 (2)	0.0350 (18)	0.0031 (15)	0.0142 (15)	0.0077 (15)
C8A	0.0251 (17)	0.036 (2)	0.0311 (17)	−0.0013 (15)	0.0076 (14)	0.0043 (15)
C9A	0.0253 (16)	0.0313 (18)	0.0225 (15)	−0.0006 (14)	0.0076 (13)	0.0005 (14)
C10A	0.0242 (16)	0.0191 (17)	0.0285 (16)	0.0002 (13)	0.0094 (13)	−0.0003 (13)
C11A	0.042 (2)	0.038 (2)	0.0353 (19)	0.0124 (17)	0.0158 (16)	0.0044 (16)
C12A	0.052 (2)	0.046 (2)	0.041 (2)	0.0273 (18)	0.0150 (18)	0.0110 (17)
C13A	0.055 (2)	0.034 (2)	0.0287 (18)	0.0092 (18)	0.0070 (16)	0.0118 (15)
C14A	0.0377 (19)	0.034 (2)	0.0285 (17)	−0.0076 (16)	0.0093 (15)	0.0004 (15)
C15A	0.0246 (17)	0.0280 (18)	0.0315 (17)	−0.0017 (14)	0.0076 (14)	0.0030 (14)
C16A	0.0210 (16)	0.0288 (18)	0.0228 (15)	0.0048 (14)	0.0065 (12)	0.0039 (14)
C17A	0.0257 (17)	0.0298 (19)	0.0428 (19)	−0.0002 (15)	0.0111 (15)	0.0011 (15)
C18A	0.0298 (19)	0.041 (2)	0.046 (2)	0.0076 (17)	0.0176 (16)	0.0000 (17)
C19A	0.0238 (17)	0.049 (2)	0.043 (2)	0.0011 (17)	0.0143 (15)	0.0089 (17)
C20A	0.0290 (19)	0.044 (2)	0.057 (2)	−0.0136 (17)	0.0135 (17)	−0.0056 (18)
C21A	0.0307 (19)	0.032 (2)	0.049 (2)	−0.0039 (16)	0.0147 (16)	−0.0089 (16)
C22A	0.0244 (17)	0.0307 (19)	0.0292 (17)	0.0050 (14)	0.0049 (13)	0.0086 (14)
C23A	0.0373 (19)	0.028 (2)	0.0436 (19)	0.0045 (16)	0.0059 (16)	0.0094 (16)
C24A	0.043 (2)	0.036 (2)	0.047 (2)	0.0094 (17)	0.0113 (17)	0.0104 (17)
C25A	0.042 (2)	0.057 (3)	0.049 (2)	0.0204 (19)	0.0174 (17)	0.0084 (18)
C26A	0.054 (3)	0.077 (3)	0.070 (3)	0.037 (2)	0.019 (2)	0.007 (2)

### Geometric parameters (Å, º)

**Table:**

Cl1—C9	1.738 (3)	C24—H24A	0.9900
Cl2—C5	1.738 (3)	C24—H24B	0.9900
Cl1A—C5A	1.740 (3)	C25—H25A	0.9900
Cl2A—C9A	1.734 (3)	C25—H25B	0.9900
N1—C3	1.390 (3)	C26—H26A	0.9800
N1—C1	1.373 (3)	C26—H26B	0.9800
N1—C22	1.456 (3)	C26—H26C	0.9800
N2—C2	1.390 (3)	C1A—C4A	1.475 (4)
N2—C1	1.318 (3)	C2A—C3A	1.371 (4)
N1A—C2A	1.392 (4)	C2A—C10A	1.459 (4)
N1A—C1A	1.313 (3)	C3A—C16A	1.486 (4)
N2A—C1A	1.367 (4)	C4A—C5A	1.392 (4)
N2A—C3A	1.383 (3)	C4A—C9A	1.398 (4)
N2A—C22A	1.471 (4)	C5A—C6A	1.383 (4)
C1—C4	1.476 (4)	C6A—C7A	1.375 (4)
C2—C3	1.373 (4)	C7A—C8A	1.379 (4)
C2—C10	1.471 (4)	C8A—C9A	1.380 (4)
C3—C16	1.484 (4)	C10A—C11A	1.390 (4)
C4—C5	1.392 (4)	C10A—C15A	1.397 (4)
C4—C9	1.397 (4)	C11A—C12A	1.377 (4)
C5—C6	1.386 (4)	C12A—C13A	1.383 (4)
C6—C7	1.379 (4)	C13A—C14A	1.377 (4)
C7—C8	1.376 (4)	C14A—C15A	1.379 (4)
C8—C9	1.381 (4)	C16A—C17A	1.382 (4)
C10—C15	1.393 (4)	C16A—C21A	1.386 (4)
C10—C11	1.386 (4)	C17A—C18A	1.386 (4)
C11—C12	1.384 (4)	C18A—C19A	1.378 (4)
C12—C13	1.381 (4)	C19A—C20A	1.372 (4)
C13—C14	1.380 (4)	C20A—C21A	1.385 (4)
C14—C15	1.377 (4)	C22A—C23A	1.518 (4)
C16—C17	1.391 (4)	C23A—C24A	1.536 (4)
C16—C21	1.383 (4)	C24A—C25A	1.507 (4)
C17—C18	1.381 (4)	C25A—C26A	1.527 (5)
C18—C19	1.376 (4)	C6A—H6A	0.9500
C19—C20	1.376 (4)	C7A—H7A	0.9500
C20—C21	1.384 (4)	C8A—H8A	0.9500
C22—C23	1.520 (4)	C11A—H11A	0.9500
C23—C24	1.524 (4)	C12A—H12A	0.9500
C24—C25	1.520 (4)	C13A—H13A	0.9500
C25—C26	1.525 (4)	C14A—H14A	0.9500
C6—H6	0.9500	C15A—H15A	0.9500
C7—H7	0.9500	C17A—H17A	0.9500
C8—H8	0.9500	C18A—H18A	0.9500
C11—H11	0.9500	C19A—H19A	0.9500
C12—H12	0.9500	C20A—H20A	0.9500
C13—H13	0.9500	C21A—H21A	0.9500
C14—H14	0.9500	C22A—H22C	0.9900
C15—H15	0.9500	C22A—H22D	0.9900
C17—H17	0.9500	C23A—H23C	0.9900
C18—H18	0.9500	C23A—H23D	0.9900
C19—H19	0.9500	C24A—H24C	0.9900
C20—H20	0.9500	C24A—H24D	0.9900
C21—H21	0.9500	C25A—H25C	0.9900
C22—H22B	0.9900	C25A—H25D	0.9900
C22—H22A	0.9900	C26A—H26D	0.9800
C23—H23B	0.9900	C26A—H26E	0.9800
C23—H23A	0.9900	C26A—H26F	0.9800
			
C1—N1—C3	106.4 (2)	H26A—C26—H26B	109.00
C1—N1—C22	126.3 (2)	C25—C26—H26A	110.00
C3—N1—C22	127.3 (2)	C25—C26—H26B	109.00
C1—N2—C2	105.6 (2)	C25—C26—H26C	109.00
C1A—N1A—C2A	105.9 (2)	N1A—C1A—N2A	111.8 (2)
C1A—N2A—C22A	127.1 (2)	N1A—C1A—C4A	123.7 (3)
C3A—N2A—C22A	125.9 (2)	N2A—C1A—C4A	124.6 (2)
C1A—N2A—C3A	106.8 (2)	N1A—C2A—C3A	109.5 (2)
N2—C1—C4	123.7 (2)	N1A—C2A—C10A	119.8 (2)
N1—C1—C4	124.3 (2)	C3A—C2A—C10A	130.7 (3)
N1—C1—N2	112.0 (2)	N2A—C3A—C2A	106.1 (2)
N2—C2—C10	119.8 (2)	N2A—C3A—C16A	121.6 (2)
C3—C2—C10	130.3 (2)	C2A—C3A—C16A	132.2 (2)
N2—C2—C3	109.9 (2)	C1A—C4A—C5A	122.0 (2)
N1—C3—C2	106.1 (2)	C1A—C4A—C9A	121.9 (2)
N1—C3—C16	122.2 (2)	C5A—C4A—C9A	116.0 (2)
C2—C3—C16	131.3 (2)	Cl1A—C5A—C4A	118.9 (2)
C1—C4—C5	122.6 (2)	Cl1A—C5A—C6A	118.6 (2)
C1—C4—C9	120.6 (2)	C4A—C5A—C6A	122.5 (3)
C5—C4—C9	116.6 (2)	C5A—C6A—C7A	119.5 (3)
Cl2—C5—C6	118.8 (2)	C6A—C7A—C8A	120.2 (3)
C4—C5—C6	122.1 (3)	C7A—C8A—C9A	119.4 (3)
Cl2—C5—C4	119.2 (2)	Cl2A—C9A—C4A	119.4 (2)
C5—C6—C7	119.1 (3)	Cl2A—C9A—C8A	118.1 (2)
C6—C7—C8	120.8 (3)	C4A—C9A—C8A	122.5 (2)
C7—C8—C9	119.1 (3)	C2A—C10A—C11A	119.1 (3)
Cl1—C9—C8	119.2 (2)	C2A—C10A—C15A	123.4 (3)
C4—C9—C8	122.3 (2)	C11A—C10A—C15A	117.4 (2)
Cl1—C9—C4	118.50 (19)	C10A—C11A—C12A	121.3 (3)
C2—C10—C11	120.0 (2)	C11A—C12A—C13A	120.7 (3)
C2—C10—C15	121.8 (2)	C12A—C13A—C14A	118.7 (3)
C11—C10—C15	118.2 (2)	C13A—C14A—C15A	120.9 (3)
C10—C11—C12	120.9 (3)	C10A—C15A—C14A	121.0 (3)
C11—C12—C13	120.3 (3)	C3A—C16A—C17A	122.5 (3)
C12—C13—C14	119.2 (3)	C3A—C16A—C21A	119.0 (3)
C13—C14—C15	120.7 (3)	C17A—C16A—C21A	118.6 (3)
C10—C15—C14	120.8 (3)	C16A—C17A—C18A	120.9 (3)
C3—C16—C17	123.0 (2)	C17A—C18A—C19A	119.9 (3)
C17—C16—C21	118.3 (2)	C18A—C19A—C20A	119.7 (3)
C3—C16—C21	118.7 (2)	C19A—C20A—C21A	120.5 (3)
C16—C17—C18	120.9 (3)	C16A—C21A—C20A	120.4 (3)
C17—C18—C19	119.9 (3)	N2A—C22A—C23A	111.5 (2)
C18—C19—C20	120.1 (3)	C22A—C23A—C24A	113.2 (2)
C19—C20—C21	119.8 (3)	C23A—C24A—C25A	114.7 (3)
C16—C21—C20	121.0 (3)	C24A—C25A—C26A	113.0 (3)
N1—C22—C23	112.2 (2)	C5A—C6A—H6A	120.00
C22—C23—C24	114.1 (2)	C7A—C6A—H6A	120.00
C23—C24—C25	114.2 (2)	C6A—C7A—H7A	120.00
C24—C25—C26	111.9 (2)	C8A—C7A—H7A	120.00
C5—C6—H6	120.00	C7A—C8A—H8A	120.00
C7—C6—H6	120.00	C9A—C8A—H8A	120.00
C8—C7—H7	120.00	C10A—C11A—H11A	119.00
C6—C7—H7	120.00	C12A—C11A—H11A	119.00
C7—C8—H8	120.00	C11A—C12A—H12A	120.00
C9—C8—H8	120.00	C13A—C12A—H12A	120.00
C10—C11—H11	120.00	C12A—C13A—H13A	121.00
C12—C11—H11	120.00	C14A—C13A—H13A	121.00
C13—C12—H12	120.00	C13A—C14A—H14A	120.00
C11—C12—H12	120.00	C15A—C14A—H14A	120.00
C14—C13—H13	120.00	C10A—C15A—H15A	119.00
C12—C13—H13	120.00	C14A—C15A—H15A	119.00
C13—C14—H14	120.00	C16A—C17A—H17A	120.00
C15—C14—H14	120.00	C18A—C17A—H17A	120.00
C10—C15—H15	120.00	C17A—C18A—H18A	120.00
C14—C15—H15	120.00	C19A—C18A—H18A	120.00
C16—C17—H17	120.00	C18A—C19A—H19A	120.00
C18—C17—H17	120.00	C20A—C19A—H19A	120.00
C19—C18—H18	120.00	C19A—C20A—H20A	120.00
C17—C18—H18	120.00	C21A—C20A—H20A	120.00
C18—C19—H19	120.00	C16A—C21A—H21A	120.00
C20—C19—H19	120.00	C20A—C21A—H21A	120.00
C21—C20—H20	120.00	N2A—C22A—H22C	109.00
C19—C20—H20	120.00	N2A—C22A—H22D	109.00
C16—C21—H21	120.00	C23A—C22A—H22C	109.00
C20—C21—H21	119.00	C23A—C22A—H22D	109.00
N1—C22—H22B	109.00	H22C—C22A—H22D	108.00
C23—C22—H22A	109.00	C22A—C23A—H23C	109.00
C23—C22—H22B	109.00	C22A—C23A—H23D	109.00
N1—C22—H22A	109.00	C24A—C23A—H23C	109.00
H22A—C22—H22B	108.00	C24A—C23A—H23D	109.00
H23A—C23—H23B	108.00	H23C—C23A—H23D	108.00
C22—C23—H23B	109.00	C23A—C24A—H24C	109.00
C24—C23—H23B	109.00	C23A—C24A—H24D	109.00
C22—C23—H23A	109.00	C25A—C24A—H24C	109.00
C24—C23—H23A	109.00	C25A—C24A—H24D	109.00
C23—C24—H24A	109.00	H24C—C24A—H24D	108.00
H24A—C24—H24B	108.00	C24A—C25A—H25C	109.00
C23—C24—H24B	109.00	C24A—C25A—H25D	109.00
C25—C24—H24A	109.00	C26A—C25A—H25C	109.00
C25—C24—H24B	109.00	C26A—C25A—H25D	109.00
C24—C25—H25B	109.00	H25C—C25A—H25D	108.00
C26—C25—H25A	109.00	C25A—C26A—H26D	109.00
C26—C25—H25B	109.00	C25A—C26A—H26E	110.00
C24—C25—H25A	109.00	C25A—C26A—H26F	110.00
H25A—C25—H25B	108.00	H26D—C26A—H26E	109.00
H26A—C26—H26C	110.00	H26D—C26A—H26F	109.00
H26B—C26—H26C	109.00	H26E—C26A—H26F	109.00
			
C3—N1—C1—N2	0.1 (3)	C11—C12—C13—C14	0.0 (5)
C3—N1—C1—C4	−179.9 (2)	C12—C13—C14—C15	0.2 (5)
C22—N1—C1—N2	−175.8 (2)	C13—C14—C15—C10	−0.1 (4)
C22—N1—C1—C4	4.2 (4)	C3—C16—C21—C20	178.8 (3)
C1—N1—C3—C2	−0.1 (3)	C3—C16—C17—C18	−179.4 (3)
C1—N1—C3—C16	173.3 (2)	C21—C16—C17—C18	0.2 (4)
C22—N1—C3—C2	175.7 (2)	C17—C16—C21—C20	−0.8 (4)
C22—N1—C3—C16	−10.9 (4)	C16—C17—C18—C19	0.8 (4)
C1—N1—C22—C23	78.7 (3)	C17—C18—C19—C20	−1.3 (4)
C3—N1—C22—C23	−96.3 (3)	C18—C19—C20—C21	0.7 (4)
C2—N2—C1—N1	0.1 (3)	C19—C20—C21—C16	0.3 (4)
C2—N2—C1—C4	−180.0 (2)	N1—C22—C23—C24	179.6 (2)
C1—N2—C2—C3	−0.1 (3)	C22—C23—C24—C25	−64.9 (3)
C1—N2—C2—C10	178.7 (2)	C23—C24—C25—C26	−166.8 (2)
C2A—N1A—C1A—N2A	0.1 (3)	N1A—C1A—C4A—C5A	104.6 (3)
C2A—N1A—C1A—C4A	−177.6 (2)	N1A—C1A—C4A—C9A	−70.9 (4)
C1A—N1A—C2A—C3A	0.6 (3)	N2A—C1A—C4A—C5A	−72.8 (4)
C1A—N1A—C2A—C10A	−178.6 (2)	N2A—C1A—C4A—C9A	111.8 (3)
C3A—N2A—C1A—C4A	177.0 (2)	N1A—C2A—C3A—N2A	−1.0 (3)
C22A—N2A—C1A—N1A	174.8 (2)	N1A—C2A—C3A—C16A	174.2 (3)
C3A—N2A—C22A—C23A	86.8 (3)	C10A—C2A—C3A—N2A	178.1 (3)
C3A—N2A—C1A—N1A	−0.7 (3)	C10A—C2A—C3A—C16A	−6.7 (5)
C1A—N2A—C22A—C23A	−88.0 (3)	N1A—C2A—C10A—C11A	−14.2 (4)
C1A—N2A—C3A—C16A	−174.8 (2)	N1A—C2A—C10A—C15A	161.4 (3)
C22A—N2A—C1A—C4A	−7.5 (4)	C3A—C2A—C10A—C11A	166.8 (3)
C1A—N2A—C3A—C2A	1.0 (3)	C3A—C2A—C10A—C15A	−17.6 (5)
C22A—N2A—C3A—C2A	−174.6 (2)	N2A—C3A—C16A—C17A	−83.2 (3)
C22A—N2A—C3A—C16A	9.6 (4)	N2A—C3A—C16A—C21A	98.1 (3)
N2—C1—C4—C5	−103.4 (3)	C2A—C3A—C16A—C17A	102.2 (4)
N1—C1—C4—C9	−107.9 (3)	C2A—C3A—C16A—C21A	−76.4 (4)
N1—C1—C4—C5	76.6 (4)	C1A—C4A—C5A—Cl1A	3.5 (4)
N2—C1—C4—C9	72.2 (4)	C1A—C4A—C5A—C6A	−175.7 (3)
N2—C2—C10—C15	−152.3 (3)	C9A—C4A—C5A—Cl1A	179.2 (2)
N2—C2—C3—N1	0.2 (3)	C9A—C4A—C5A—C6A	0.0 (4)
N2—C2—C3—C16	−172.4 (3)	C1A—C4A—C9A—Cl2A	−5.9 (4)
C10—C2—C3—N1	−178.5 (3)	C1A—C4A—C9A—C8A	175.0 (3)
C10—C2—C3—C16	8.9 (5)	C5A—C4A—C9A—Cl2A	178.4 (2)
N2—C2—C10—C11	25.4 (4)	C5A—C4A—C9A—C8A	−0.8 (4)
C3—C2—C10—C11	−156.0 (3)	Cl1A—C5A—C6A—C7A	−178.8 (2)
C3—C2—C10—C15	26.3 (4)	C4A—C5A—C6A—C7A	0.4 (4)
N1—C3—C16—C17	70.2 (4)	C5A—C6A—C7A—C8A	−0.2 (4)
C2—C3—C16—C21	62.2 (4)	C6A—C7A—C8A—C9A	−0.6 (4)
N1—C3—C16—C21	−109.4 (3)	C7A—C8A—C9A—Cl2A	−178.1 (2)
C2—C3—C16—C17	−118.2 (3)	C7A—C8A—C9A—C4A	1.1 (4)
C1—C4—C5—C6	174.5 (3)	C2A—C10A—C11A—C12A	175.1 (3)
C1—C4—C5—Cl2	−4.8 (4)	C15A—C10A—C11A—C12A	−0.7 (4)
C5—C4—C9—C8	2.0 (4)	C2A—C10A—C15A—C14A	−176.3 (3)
C1—C4—C9—Cl1	8.0 (4)	C11A—C10A—C15A—C14A	−0.7 (4)
C9—C4—C5—Cl2	179.5 (2)	C10A—C11A—C12A—C13A	1.9 (5)
C9—C4—C5—C6	−1.3 (4)	C11A—C12A—C13A—C14A	−1.6 (5)
C1—C4—C9—C8	−173.8 (3)	C12A—C13A—C14A—C15A	0.3 (4)
C5—C4—C9—Cl1	−176.2 (2)	C13A—C14A—C15A—C10A	0.9 (4)
Cl2—C5—C6—C7	179.1 (2)	C3A—C16A—C17A—C18A	179.6 (3)
C4—C5—C6—C7	−0.2 (4)	C21A—C16A—C17A—C18A	−1.8 (4)
C5—C6—C7—C8	1.0 (4)	C3A—C16A—C21A—C20A	−179.7 (3)
C6—C7—C8—C9	−0.3 (4)	C17A—C16A—C21A—C20A	1.7 (4)
C7—C8—C9—C4	−1.3 (4)	C16A—C17A—C18A—C19A	0.7 (4)
C7—C8—C9—Cl1	176.9 (2)	C17A—C18A—C19A—C20A	0.7 (4)
C11—C10—C15—C14	−0.3 (4)	C18A—C19A—C20A—C21A	−0.8 (4)
C2—C10—C15—C14	177.4 (3)	C19A—C20A—C21A—C16A	−0.4 (4)
C2—C10—C11—C12	−177.2 (3)	N2A—C22A—C23A—C24A	−175.6 (2)
C15—C10—C11—C12	0.5 (4)	C22A—C23A—C24A—C25A	63.0 (3)
C10—C11—C12—C13	−0.3 (5)	C23A—C24A—C25A—C26A	168.4 (2)

### Hydrogen-bond geometry (Å, º)

Cg1 is the centroid of the N1/N2/C1–C3 imidazole ring.

**Table:**

*D*—H···*A*	*D*—H	H···*A*	*D*···*A*	*D*—H···*A*
C12*A*—H12*A*···Cl1^i^	0.95	2.70	3.512 (4)	143
C21—H21···*Cg*1^ii^	0.95	2.88	3.734 (3)	151

Symmetry codes: (i) *x*, *y*, *z*+1; (ii) −*x*+1, −*y*, −*z*+1.
